# Enhanced Path Planning and Obstacle Avoidance Based on High-Precision Mapping and Positioning

**DOI:** 10.3390/s24103100

**Published:** 2024-05-13

**Authors:** Feng Zhang, Leijun Li, Peiquan Xu, Pengyu Zhang

**Affiliations:** 1 School of Materials Science and Engineering, Shanghai University of Engineering Science, Shanghai 201620, China; m350121322@sues.edu.cn; 2 Department of Chemical and Materials Engineering, University of Alberta, Edmonton, AB T6G 1H9, Canada; leijun@ualberta.ca; 3 Shanghai Collaborative Innovation Center of Laser Advanced Manufacturing Technology, Shanghai University of Engineering Science, Shanghai 201620, China; 4 School of Mechatronic Engineering and Automation, Shanghai University, Shanghai 201900, China

**Keywords:** cartographer algorithm, iterative closest point (ICP), inspection robot, TEB algorithm

## Abstract

High-precision positioning and multi-target detection have been proposed as key technologies for robotic path planning and obstacle avoidance. First, the Cartographer algorithm was used to generate high-quality maps. Then, the iterative nearest point (ICP) and the occupation probability algorithms were combined to scan and match the local point cloud, and the positions and attitudes of the robot were obtained. Furthermore, Sparse Matrix Pose Optimization was carried out to improve the positioning accuracy. The positioning accuracy of the robot in x and y directions was kept within 5 cm, the angle error was controlled within 2°, and the positioning time was reduced by 40%. An improved timing elastic band (TEB) algorithm was proposed to guide the robot to move safely and smoothly. A critical factor was introduced to adjust the distance between the waypoints and the obstacle, generating a safer trajectory, and increasing the constraint of acceleration and end speed; thus, smooth navigation of the robot to the target point was achieved. The experimental results showed that, in the case of multiple obstacles being present, the robot could choose the path with fewer obstacles, and the robot moved smoothly when facing turns and approaching the target point by reducing its overshoot. The proposed mapping, positioning, and improved TEB algorithms were effective for high-precision positioning and efficient multi-target detection.

## 1. Introduction

With the development of artificial intelligence technology, autonomous inspection robots have become an integral part of various industries, including manufacturing, consumer services, and healthcare [1]. These robots perform tasks in complex, hazardous, or hard-to-reach environments, reducing labor costs, improving efficiency, and mitigating risks associated with the work. Autonomous inspection robots are significant in modern industry across various sectors. To achieve customized inspection functionalities, they should possess the basic capability to identify and navigate around obstacles [2]. Additionally, high-precision localization and path planning abilities are necessary for accurate task completion and efficient operations.

Researchers have been working to improve mapping, localization accuracy, and optimize path planning [3]. Simultaneous Localization and Mapping (SLAM) provides a solution for robot localization by estimating the robot’s position in unknown environments while mapping the surroundings. Visual SLAM (vSLAM) methods, such as Mono-SLAM [4], PTAM [5], ORB-SLAM [6], ORB-SLAM2 [7], and OpenVSLAM [8], have been extensively studied for cost-effective indoor localization. In reference [9], VGG16-based image descriptors are used for robust image retrieval. The Top-N similar images are extracted, and candidate images are reordered using region descriptors based on Faster RCNN. Laser-based SLAM algorithms, such as Gmapping [10] and Hector SLAM [11], offer higher accuracy and faster computation speeds.

However, both of these Bayesian-based methods lack cycle detection and optimization, and they require time-consuming pre-mapping of the entire environment to construct a high-resolution geographic feature database for precise localization. It is challenging to achieve continuous tracking of a robot in dynamic indoor and outdoor environments by detecting key frames. The approach of frequently building visual feature databases to address this problem is highly inefficient. Instead, graph-based mapping software [12] can accurately and robustly create maps. Laser-based SLAM methods can also be used for global localization of robots. Matching algorithms [13,14] match the query scans with a key point database generated from grid-based maps, retrieving relevant position information to achieve high-precision robot localization. Other probabilistic localization algorithms [15,16,17,18] change the probability of the robot pose through motion and observation models, but discrepancies between the constructed map and the physical environment may result in inaccuracies.

The planning of inspection robot paths involves two stages: global path planning and local path planning. This paper focuses on local path planning [19]. In real-world scenarios where humans and robots coexist, the robot’s perception of dynamic obstacles in the surroundings is weak, even when it has basic global map information. Therefore, selecting and optimizing local path planning is crucial for achieving robot motion in complex and dynamic inspection spaces. Common local path planners include artificial potential fields [20], reactive replanning methods [21,22], and fuzzy algorithm-based methods [23]. Artificial potential fields are relatively simple and exhibit good real-time planning performance. However, traditional artificial potential field methods tend to fall into local optima and encounter reachability issues when obstacles surround the target point. This paper adopts the improved TEB algorithm for local path planning. Roesmann et al. proposed the TEB algorithm [24] as an extension of the classical EB algorithm. This algorithm optimizes multiple objectives to provide obstacle avoidance through trajectory optimization. Unlike local path planning methods, the TEB algorithm can set multiple constraint conditions as needed to ensure its applicability. However, mobile robots equipped with the TEB algorithm may get stuck in local minima while navigating complex environments, making it difficult to traverse obstacles. To address this issue, Rösmann et al. proposed extensions of the TEB technique by using parallel trajectory planning within spatially unique topological structures [25,26]. However, these methods only consider the positions of obstacles and do not account for potential collisions between the robot and surrounding obstacles. Lan et al. proposed an Active Timed Elastic Band (PTEB) technique for autonomous mobile robot navigation systems in dynamic environments [27]. Previous efforts to improve the performance of the TEB algorithm in complex environments have mainly focused on obstacle avoidance. Additionally, most related re-search aims to avoid local minima and achieve smooth planning paths for AGVs in complex environments, lacking constraints on the shortest local path and resulting in non-optimal local path planning [28]. 

To achieve high-precision positioning and efficient multi-target detection in inspection robots, we employed the Cartographer algorithm to construct high-quality maps and enhanced the scanning matching module for precise localization. Leveraging these maps and localization modules, we refined the Time-Elastic Band (TEB) algorithm by incorporating critical coefficients to adjust the distance between the robot and obstacles, enhancing the robot’s operational safety. Additionally, constraints on acceleration and terminal velocity were introduced to ensure smooth velocity transitions during motion and as the robot approaches targets.

The primary contributions of this study are summarized as follows:

(1) We improved the scanning matching module by integrating the Iterative Closest Point (ICP) algorithm with occupancy probability methods, constructing Euclidean submaps to determine the robot’s positioning pose. The optimal pose was then refined using a sparse matrix for pose optimization.
(2) Building on precise localization, we enhanced the traditional TEB algorithm by introducing critical coefficients and adding constraints on acceleration and terminal velocity to boost the robot’s safety and the smoothness of its movements.

## 2. Materials and Methods

### 2.1. Cartographer High-Precision Mapping Process

The Cartographer algorithm has improved the mapping algorithm based on particle filter method by reducing memory consumption in large-scale environments. It adopts a backend data processing method based on graph optimization to construct two-dimensional or three-dimensional environment maps. The algorithm changes the way of constructing global maps from directly using sensor data to first generating submaps and then indirectly constructing global maps. 

**Figure 1:**
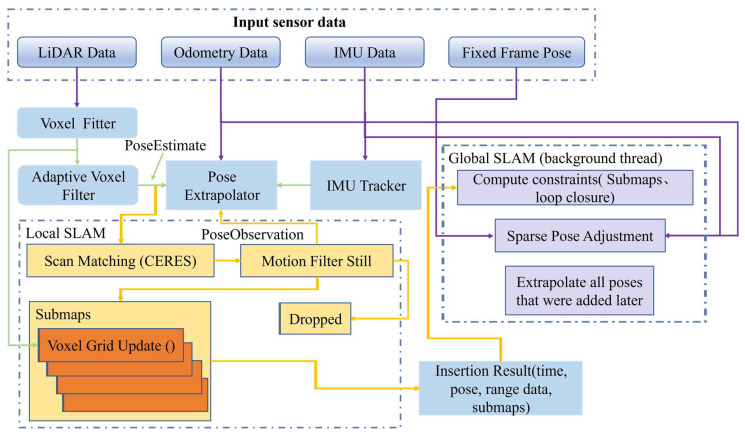
The flowchart of the Cartographer algorithm.

After generating all the submaps, the Cartographer algorithm applies backend pose graph optimization to correct errors. Therefore, the environment maps established using the Cartographer algorithm have a lower level of error. The mapping algorithm framework can be divided into three parts: receiving sensor data, local SLAM, and global SLAM. The algorithm’s mapping framework is illustrated in Figure 1.

During the mapping process, laser scan observations, odometer data, and IMU data are fused together. In the local SLAM process, newly acquired laser scan data is matched with nearby submaps to find the optimal insertion pose and insert the scan data into the submap. However, errors can accumulate during the scan matching process, and these errors can accumulate over time and as the map grows. In the global SLAM process, pose graph optimization adjusts the robot’s poses to eliminate errors, thus constructing a globally consistent map.

Submaps are built during the local SLAM process. To enhance map accuracy, the Cartographer algorithm performs pose optimization on the data before inserting laser scan point cloud data into the submaps. This is achieved through Ceres nonlinear optimization, refining the robot’s pose through scan matching to increase the likelihood of point cloud matching within the submaps. Once the submaps are constructed, a global map can be derived from them.

### 2.2. Robot Localization Based on Scan Matching

#### 2.2.1. ICP Algorithm

Scan matching is employed to align the current laser scan data with previous map data to estimate the robot’s pose. The algorithm iteratively reduces the error between scan data and map data. The robot’s pose is updated based on the optimized results until the optimal robot pose is obtained.

**Figure 2:**
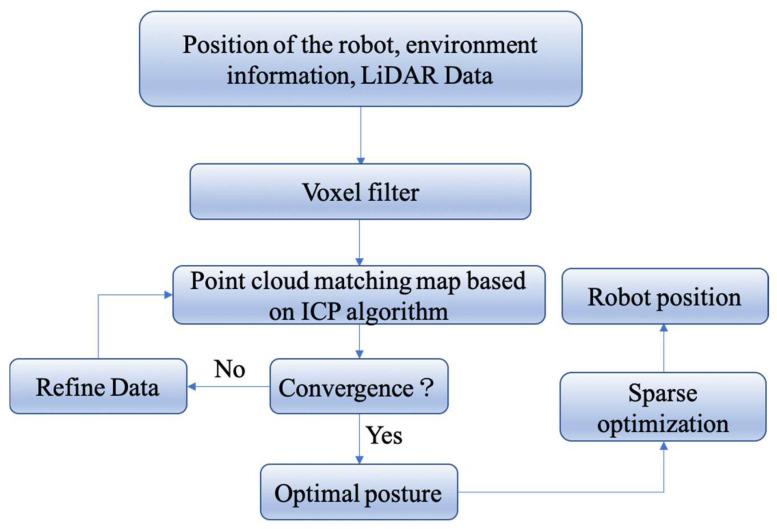
Schematic diagram of the localization method.

The ICP-based optimization algorithm is a method that applies voxel filters in the point cloud library (PCL) to reduce the noise in the point cloud [29]. The ICP algorithm matches the scan to the submap for point cloud registration, and convergence is checked to determine if the algorithm has converged to the best matching pose. If convergence is achieved, the robot’s position is updated, and the process is repeated. Otherwise, further iteration or data optimization may be necessary before repeating the process. This iterative process determines the optimal pose, and the obtained optimal pose undergoes sparse matrix pose optimization to finalize the robot’s position information. The robot’s localization framework is illustrated in Figure 2.

**Figure 3:**
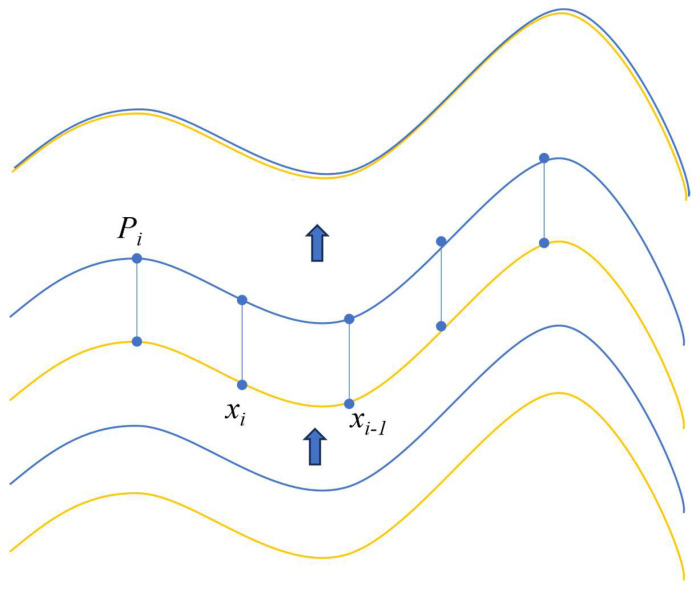
Principle of the ICP algorithm.

In laser SLAM for mobile robot localization, it is challenging to rely on wheel encoders to obtain mileage information or rely on inertial navigation systems to obtain position data. ICP-based approach is an effective solution. This method utilizes laser ranging technology to reconstruct the robot’s motion state during motion by means of adjacent laser point cloud pairs. The ICP employs a point cloud matching model for inter-frame alignment, uses the nearest neighbor principle to locate candidate points for matching, and constructs a cost function based on these matches as shown in Equation (1). By searching for the best matching parameters and, the position information of the robot can be obtained.

(1) $$e=\arg\underset{\delta }{min}\;\sum_{i=1}^{N}\;\|\delta ⊕p_{k}-q_{j}{\|}^{2}$$

where N represents the number of point cloud, and the reference frame, and $q_{j}$ represents the set of reference frame point clouds. The undulating lines depicted in Figure 3 serve as visualizations of obstacle surfaces. Within this context, $P_{i}$ signifies a point awaiting matching, while $x_{i}$ denotes a point extracted from the point cloud of the preceding frame. The objective lies in matching each point $x_{i}$ with its closest neighbor $P_{i}$.

#### 2.2.2. Improved ICP Matching Model

However, this approach is time-consuming, and the robot’s current position may change when the iteration is completed. Moreover, the approach involves substantial computational overhead, leading to significant cumulative errors during matching, which may decrease the precision of robot localization, making real-time localization challenging.

To solve the above problems, this paper uses a combination of ICP algorithm and occupancy probability method to construct Euclidean subgraphs to improve the robot’s localization accuracy. The ICP algorithm is used to iteratively align the point cloud with the previously known errors of the map information to continuously reduce the errors. The optimization model is shown in Equation (2):

(2) $$\min\;\sum_{i=1}^{n}\;∥p_{i}-(Rq_{i}+t){∥}^{2}$$

where $p_{i}$ is the points in the current point cloud (from a known map), $q_{i}$ is the data from the contingent sensors. R is the rotation matrix and t is the translation vector.

The occupancy probability approach is modeled as in Equation (3). where o denotes the occupancy state (occupied/unoccupied), z denotes the sensor measurements, and *x* denotes the robot’s bit position. The occupancy probability *P*; given the sensor measurements and the robot’s bit position adjusts the point cloud according to the possible obstacles.

(3) $$P_{0}=P(o|z,x)$$

The Euclidean distance subgraph is created based on the above principle as in Equation (4), where the acquired point cloud is point $p_{i}$, and $e_{ij}$ represents the distance between *p_i_* and its nearest obstacle, and $o_{j}$ denotes the position of the nearest obstacle pointing to $p_{i}$.

(4) $$e_{ij}=∥p_{i}-o_{j}∥$$

To optimize the position of the point cloud with respect to obstacles, the distance from the point cloud to the nearest obstacle is minimized to effectively update the robot’s understanding of its surroundings. Combining Equations (2) and (4) defines the optimal model shown in Equation (5):

(5) $$J=\underset{R,t}{min}\;\sum_{i}\;\underset{j}{min}\;∥p_{i}-(Ro_{j}+t){∥}^{2}\;$$

where J is the objective function. The updated point cloud is used to optimize the estimation of the robot’s position in its environment, thus improving the localization accuracy. The new position is updated based on minimizing the difference between the estimated position and the position collected from the sensor data as shown in Equation (6):

(6) $$x^{\prime }=\arg\underset{x}{min}\;∥F(x)-z{∥}^{2}$$

where F(x) denotes the theoretical measurement values at attitude x and z is the actual measurement values.

To obtain the global optimal solution with high precision, the collected pose information is nonlinearly optimized. The pose map is composed of the pose points with nonlinear constraints during the robot’s movement, and these constraints are also common observations around the pose points. In order to achieve high-precision positioning of the mobile robot in the actual process, LM (Levenberg-Marquardt) is used as the framework for the constraints between different poses, and the robot poses are processed by sparse matrix. This method is similar to the sparse cluster optimization problem of camera sensor and external environment processed by LM in visual SLAM. Finally, the method of solving linear system by direct sparse Jorisky decomposition is adopted to deal with the optimization problem of 2D pose map for pose optimization and location information determination.

### 2.3. Localized Path Planning

#### 2.3.1. TEB Algorithm

The basic principle of the TEB algorithm is to define the path as an elastic band that connects the starting point and the target detection point, and constraints such as obstacles on the path will deform this elastic band. The expression of the TEB algorithm is shown in Equation (7):

(7) $$B=\left(Q,\tau \right)=\left\{s_{1},\Delta T_{1},s_{2},\Delta T_{2},\ldots ,s_{n-1},\Delta T_{n-1},s_{n}\right\}$$

The TEB algorithm first performs subsequent corrections based on the initial trajectory generated by the global path planner. During the correction process, the algorithm formulates the trajectory optimization problem as a multi-objective optimization problem, and these objectives are considered together in the optimization process to find the optimal trajectory. Based on different optimization objectives, m optimization functions $f_{k}(B)$ are established. The weight of each optimization function is represented as $\eta_{k}$, expressed as follows:

(8) $$f\left(B\right)=\sum \eta_{k}f_{k}\left(B\right),k=1,2,\ldots ,m$$

With fixed initial and target poses, the optimal trajectory set $B^{*}$ is obtained by solving the aforementioned optimization functions.

(9) $$B^{*}=arg_{B}minf\left(B\right)$$

In the TEB algorithm, path-following constraints can fuse the local optimization trajectory of TEB with the global path. The closest distance between pose points and global path points is defined as $d_{min,j}$, with the maximum allowable distance denoted as $r_{P_{max}}$. The penalty function is formulated as follows:

(10) $$f_{path}=e_{\tau }\left(d_{min,j},r_{P_{max}},\varepsilon ,S,n\right)$$

The obstacle avoidance constraint is applied to ensure that the optimized trajectory avoids obstacles. The minimum allowable value is set such that the distance between the pose point and the obstacle is $r_{O_{min}}$. The penalty function is formulated as follows:

(11) $$f_{ob}=e_{\tau }\left(-d_{min,j},-r_{O_{min}},\varepsilon ,S,n\right)$$

In the above two equations, S represents the deformation factor, where the polynomial coefficient n is typically set to 2. The offset factor *ε* represents a small displacement near the boundary, providing a margin for inequality constraints. The gradient of the penalty function can be interpreted as an external force acting on the elastic band.

The dynamics constraints of a mobile robot consist of the average linear and angular velocities of the robot, and a penalty function can be used to represent the constraints on linear and angular velocities. In the penalty function, the velocity can be penalized for exceeding the constraints as shown in Equation (12).

(12) $$\left\{\begin{array}{c} f_{vi}=e_{\tau }\left(v_{i},v_{max},\varepsilon ,S,n\right)\\ f_{wi}=e_{\tau }\left(w_{i},w_{max},\varepsilon ,S,n\right) \end{array}\right.$$

Similarly, the penalty functions for linear acceleration and angular acceleration are as follows:

(13) $$\left\{\begin{array}{c} f_{a_{i}}=f_{a_{i}}\left(S_{i},S_{i+1},S_{i+2},\Delta T_{i},\Delta T_{i+1}\right)\\ f_{\alpha_{i}}=f_{\alpha_{i}}\left(S_{i},S_{i+1},S_{i+2},\Delta T_{i},\Delta T_{i+1}\right) \end{array}\right.$$

The focus of this paper is to improve the constraints of the TEB algorithm. Therefore, the subsequent content will not be described further.

#### 2.3.2. Improved TEB Algorithm

**Figure 4:**
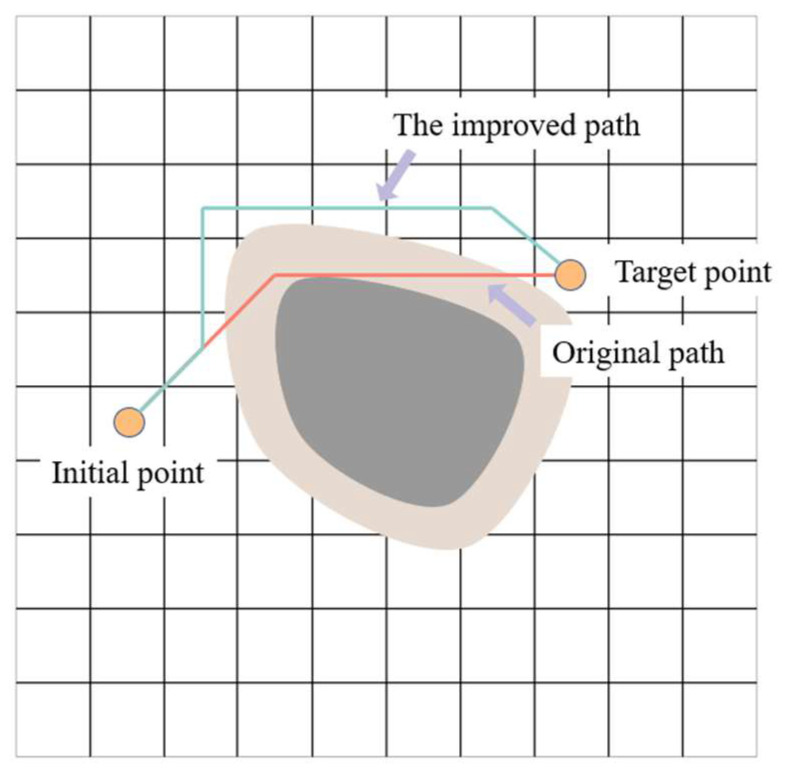
Comparison of paths under different critical coefficients.

In this improvement, a critical coefficient is introduced to adjust the distance between path points and obstacles in path planning, as shown in Figure 4. This can be used to enhance the safety and efficiency of path planning. By controlling the minimum safe distance between path points and obstacles in the path planning algorithm, it can be adjusted based on specific applications and requirements to ensure that the generated path approaches the target point as closely as possible without colliding with obstacles. This can be represented mathematically as:

(14) $$S=|P_{path}-O_{ob}|-C_{ob}\cdot K$$

where $P_{path}$ represents the position of the path point, $O_{ob}$ represents the position of the geometric center of the obstacle, $|P_{path}-O_{ob}|$ denotes the Euclidean distance between the path point and the Geometric center of the obstacle, $C_{ob}$ represents the radius of the obstacle, and *K* is the critical coefficient. Where S represents the distance between the path and the obstacle; when S is greater than half of the robot width W, the robot can pass through the obstacle region, so a reasonable range of K can be solved.

To improve the motion accuracy of the robot, acceleration and deceleration control is performed between the start-stop and corner program segments. Acceleration and deceleration control is important for path planning. If the acceleration and deceleration control is not sufficient, there will be a sudden change in acceleration/deceleration, resulting in impacts, and even the robot will not be able to stop in time when it reaches the target point, and it will cross the target point. 

The acceleration is constrained by assuming $∆X_{i},\;\;∆X_{i+1},\;\;∆X_{i+2},\;\;∆X_{i+3}$ are four consecutive positional points in the local path and the time interval between the four positional points is $∆T_{j},\;\;∆T_{j+1},\;\;∆T_{j+2}$, respectively. The linear acceleration $a_{v-t}$ and angular acceleration $a_{w-t}$ are obtained from Equations (15) and (16), respectively:

(15) $$a_{v\_t}=\frac{2(v_{t+1}-v_{t})}{∆T_{t}+∆T_{t+1}}$$

(16) $$a_{w\_t}=\frac{2(w_{t+1}-w_{t})}{∆T_{t}+∆T_{t+1}}$$

The linear acceleration and the angular acceleration constraints are derived from Equations (17) and (18):

(17) $$j_{lim\_t}=\frac{a_{v\_t+1}-a_{v\_t}}{0.25∆T_{t}+0.5∆T_{t+1}+0.25∆T_{t+2}}$$

(18) $$j_{rot\_t}=\frac{a_{w\_t+1}-a_{w\_t}}{0.25∆T_{t}+0.5∆T_{t+1}+0.25∆T_{t+2}}$$

Furthermore, it is proposed to intelligently adjust the end velocity of the robot to achieve smooth deceleration and precise arrival at the target point, thereby reducing the impact on the robot and improving the accuracy of velocity planning. This improvement contributes to enhancing the robot’s control performance and safety. Mathematically, this can be represented as:

(19) $$v_{dmax}=\left\{\begin{array}{c} v_{max}\;|{Pose}_{cur}-T|>d_{threshold}\\ \frac{d}{d_{threshold}}\times v_{max}\;|{Pose}_{cur}-T|⩽d_{threshold} \end{array}\right.$$

where $v_{max}$ is the pre-set maximum linear velocity of the robot, $d_{threshold}$ is the pre-set threshold of the Euclidean distance between the robot and the target point, $|{Pose}_{cur}-T_{\;}|$ represents the Euclidean distance between the current position of the robot and the target point, and $v_{max}$ is the maximum linear velocity of the robot during motion.

## 3. Experiment Process and Result Analysis

### 3.1. Experimental Platform and Environment

**Figure 5:**
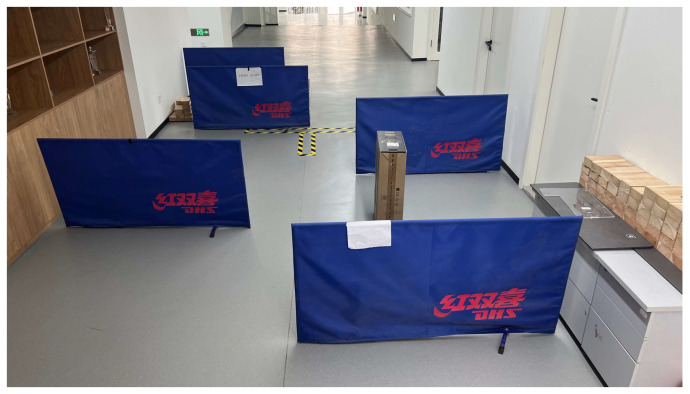
Experimental environment.

The simulated inspection environment is depicted in Figure 5. It measured 7 m in length and 3.5 m in width. The environment primarily consisted of narrow spaces in which the robot was expected to navigate around and into confined areas, as well as perform obstacle avoidance tasks. This environment could be used to conduct a diverse array of tests, such as evaluating emergency obstacle avoidance capabilities and assessing the smoothness of the robot’s velocity. The obstacles in the environment included 0.5 m × 0.8 m frames and cardboard boxes.

**Figure 6:**
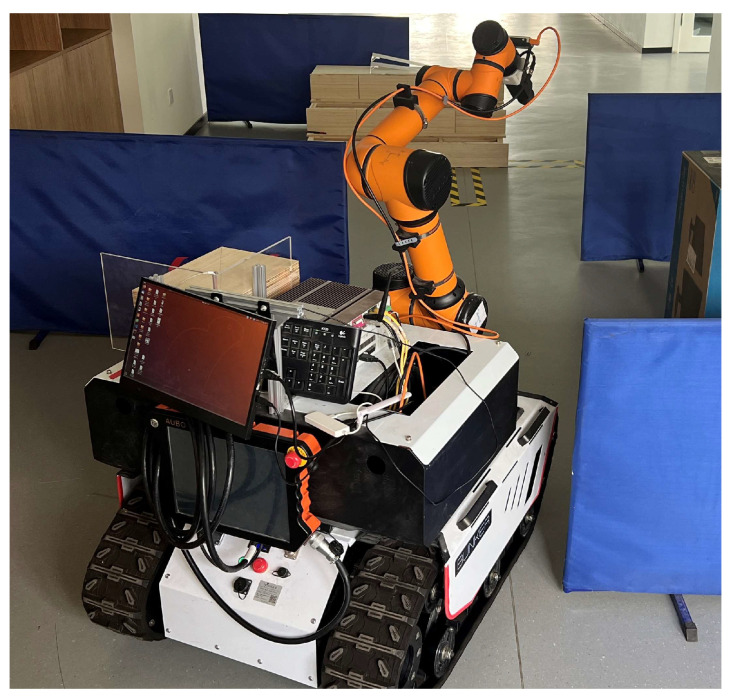
Experimental platform.

The experimental platform used a tracked differential composite robot, which is shown in Figure 6. The main control board uses an industrial computer (i7 16G 256SSD) as the controller, the LiDAR is VLP 16, and the system adopts the Ubuntu18.04 platform and integrates the ROS system. In the actual experiments, remote control was realized through the ROS distributed framework on the same LAN, which provided a stable operation environment and software support for the robot application. The main parameters of robot movement are shown in Table 1.

### 3.2. Experimental Results

#### 3.2.1. Robot Localization Accuracy Test

**Figure 7:**
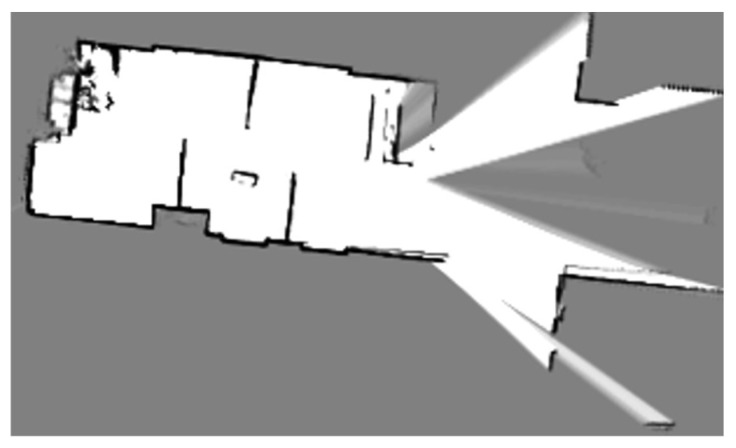
The mapping effect created from the Cartographer algorithm.

To fully demonstrate the accuracy of the mapping and localization methods, we conducted experiments using a real-world workflow that required a high level of precision. Firstly, we utilized the Cartographer algorithm to map the simulated environment, allowing the robot to perceive the surrounding obstacles. The mapping result is shown in Figure 7.

**Figure 8:**
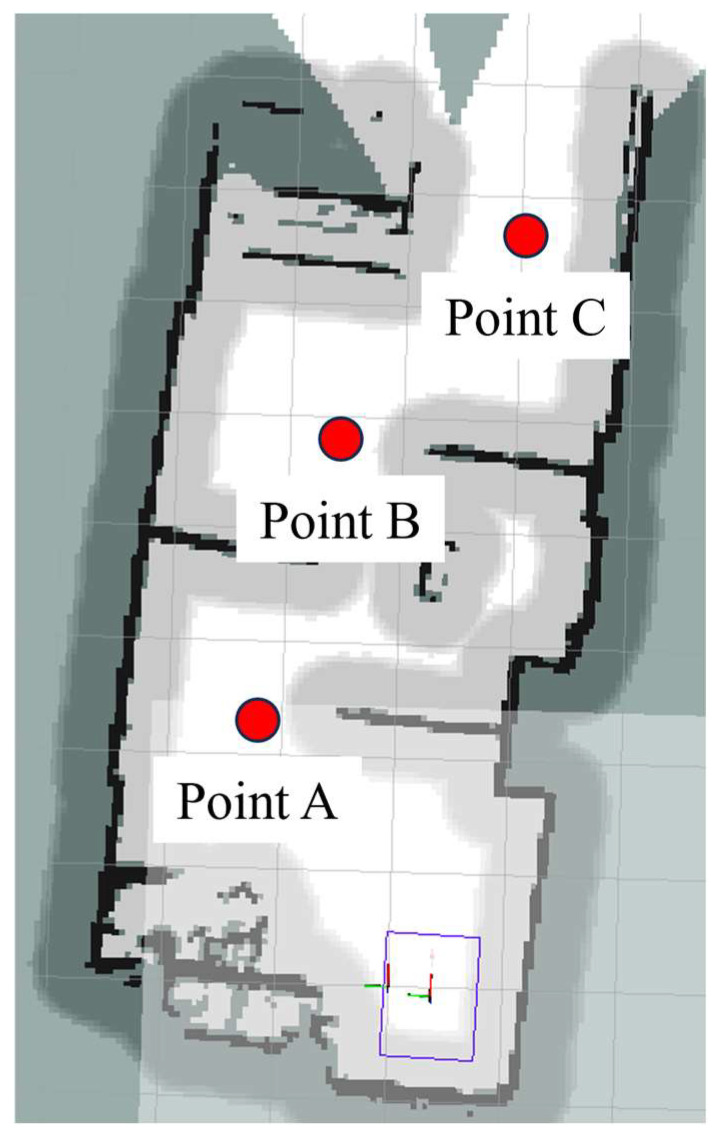
A schematic diagram of robot inspection positions.

Based on the constructed map, three inspection points, A, B, and C, required by the robot are marked as shown in the Figure 8.

**Figure 9:**
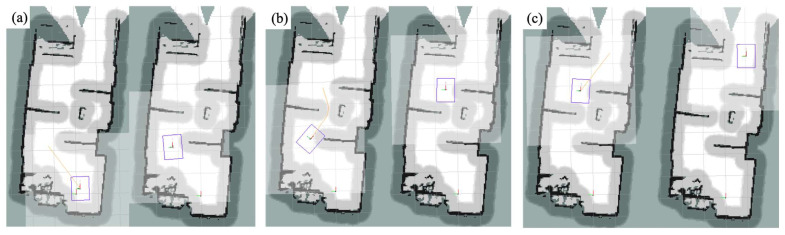
The movement process of the robot. (**a**–**c**) is the position of the robot as it passes through points A, B, and C, respectively.

In this experiment, the robot was set to move autonomously from the starting point to the target point, and three passing points, A, B, and C, were set during the movement. The robot stayed for five seconds each time it passed, and the x, y, and Angle values of the starting point were manually measured. A, B, and C do the same work. The experiment was repeated 16 times, and the measurement data from passing through the three points A, B and C were recorded each time, and the data was compared with the data from the first time, so as to judge the positioning error of the robot in the complex environment. The robot motion process is shown in Figure 9a–c.

**Figure 10:**
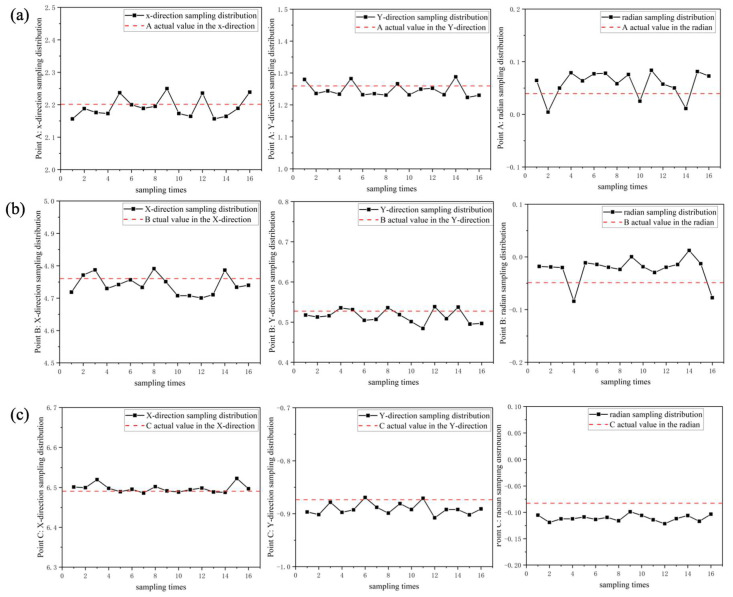
Analysis of position recording for points (**a**) A, (**b**) B, and (**c**) C.

Finally, an error analysis of the real-time position and the actual positions of three inspection points was conducted to determine the robot’s localization precision. The experimental results are depicted in Figure 10. From the graph, it is evident that the errors in the robot’s movement from the starting point to inspection points A (Figure 10a), B (Figure 10b), and C (Figure 10c) are uniformly distributed. The errors in the X and Y directions remain stable within 5 cm, while the robot’s turning radius remains stable at 0.04 radians, equivalent to 2 degrees. Such testing precision enhances the performance, ensuring that the robot can complete high-precision inspections without colliding with obstacles. 

After the drawing was completed, the positioning time before and after the improvement of the positioning algorithm was compared. The test method included turning on the LiDAR, then turning on the map and positioning algorithm, next operating the robot to implement the positioning process, and lastly recording the time of matching the robot and the map. Ten experiments were repeated to average the results. The experimental results are shown in Table 2. The efficiency of the improved positioning algorithm was increased by 40% and the positioning time of the robot was saved.

#### 3.2.2. Local Path Improvement Test

To verify the effectiveness of the improved TEB planning algorithm, this paper conducted practical experimental tests. We verified the performance of the improved TEB algorithm in two aspects: firstly, the effectiveness of planning a safe trajectory amid dense obstacles; secondly, the effectiveness of keeping the speed of the robot smooth during the movement, and arriving at the target point smoothly and accurately.

**Figure 11:**
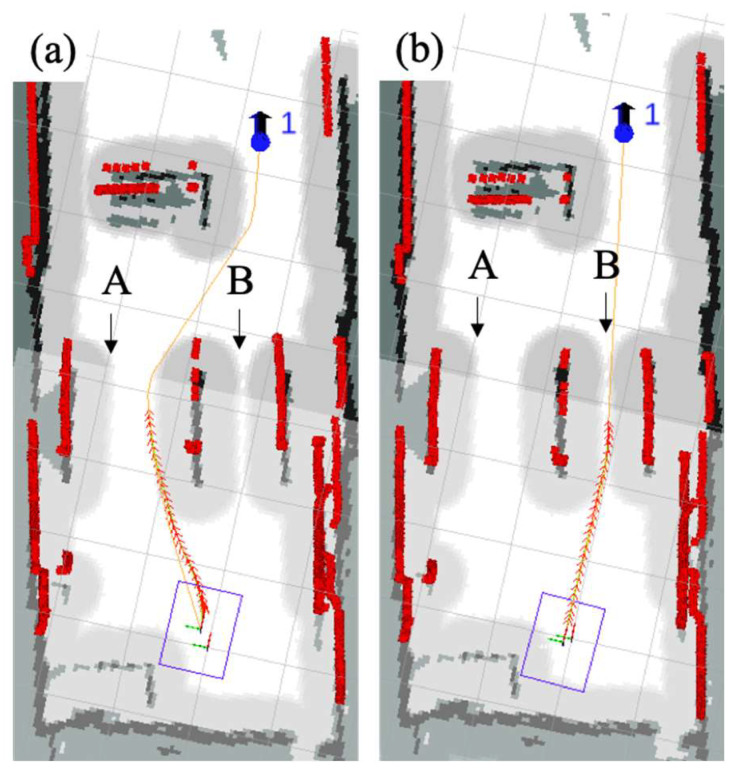
Trajectory safety analysis. (**a**) The robot passing through safe zone A, (**b**) The robot passing through dangerous zone B.

In the experimental environment, shown in Figure 11, we set up a wider channel A and a narrower obstacle channel B. The traditional TEB algorithm would choose channel B in Figure 11, but this would lead to the robot being too close to the obstacle when passing through the obstacle, which would result in a higher risk of rubbing against the obstacle. The improved TEB algorithm introduced a criticality coefficient to adjust the distance between the path points and the obstacles in the path planning, and chose the wider channel A in Figure 11, which was more favorable for safe passage of the robot. The experimental results verify the effectiveness of the improved TEB algorithm in selecting safe trajectories.

**Figure 12:**
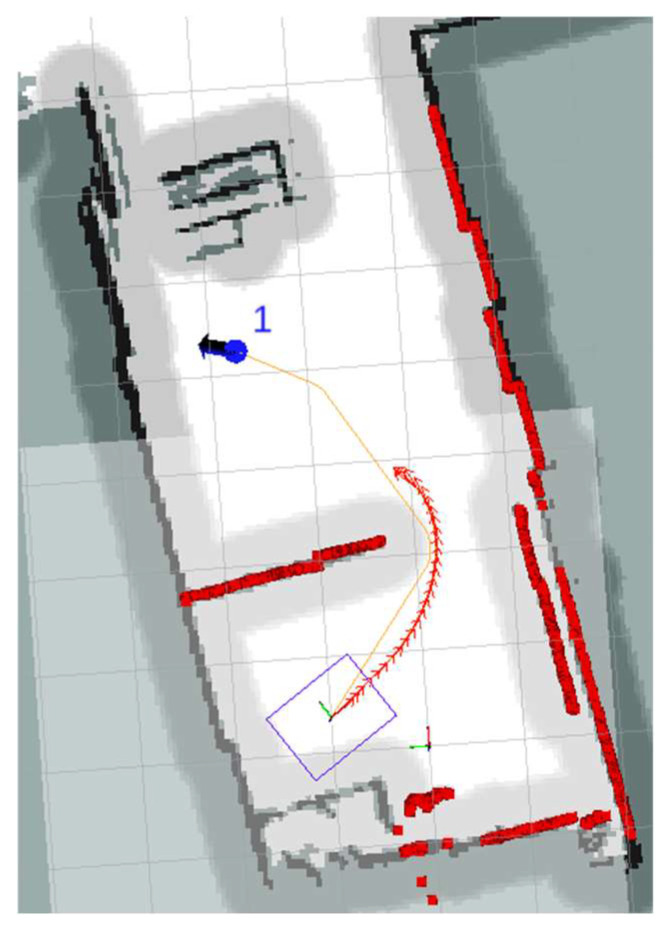
Speed-smoothing experimental environments.

By smoothing the speed of the robot, we can perform the inspection task more safely and efficiently. To validate the effectiveness of our improvements to the robot’s acceleration and end-velocity constraints, we designed an experimental environment, as shown in Figure 12. 

**Figure 13:**
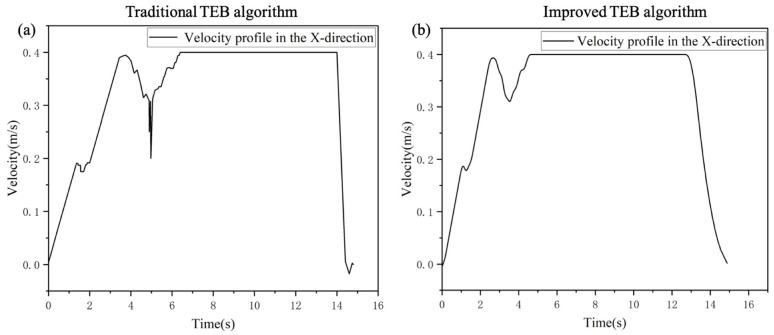
The velocity curve of the robot in the X direction (**a**) before the improvement of the TEB algorithm and (**b**) after the improvement of the TEB algorithm.

The robot started from the starting point, bypassed obstacles, and reached the final goal point. As shown in Figure 13, before the algorithmic improvement, the robot’s velocity oscillated significantly when going around the obstacle, whereas after the algorithmic improvement the robot effectively reduced the sudden change of velocity when going around the obstacle which, in turn, reduced the energy consumption during the task execution. This will extend the battery life, reduce wear and tear on the robot components, and extend the overall life of the robot. In addition, a smooth velocity profile stabilizes the robot’s motion and reduces collisions with obstacles due to velocity changes. As a result, this can improve the quality of sensor data acquisition and positioning accuracy. In practical applications, a smooth velocity profile can also shorten the robot’s emergency response time in unforeseen situations, thus improving the safety of the inspection task.

When approaching the target point, as shown in Figure 13a, the slope of the velocity curve in the traditional algorithm is suddenly changed, indicating that the absolute value of the acceleration was suddenly increased. The robot, thus, had a velocity jump, which produced a sudden stop when the robot arrived at the target point, and generated a large impact on the equipment it carried. The impact may result in the robot not accurately stopping at the target point; instead, it may perform a reverse adjustment. In contrast, as in Figure 13b the improved algorithm ran more smoothly during deceleration, the absolute value of acceleration gradually decreased to 0, and finally stopped smoothly at the target point. By comparing and analyzing these velocity curves, the significant effect of the optimization algorithm in smoothing the robot’s velocity can be clearly seen. It verifies the algorithm improvement.

## 4. Conclusions

The proposed algorithm exhibited robust strength in adapting to complex environments; it combined the ICP and occupancy probability algorithms to enhance the localization accuracy of the robot. On complex indoor terrains, the algorithm could efficiently construct maps, plan paths, and provide reliable support for robot autonomous navigation. The Cartographer algorithm was adopted to create maps through combining laser sensor data with wheel odometer and IMU data. To obtain the robot position, LiDAR was utilised to collect environmental point cloud data, and the voxel filter in PCL was used to process the environmental point cloud data in order to reduce the impact of scattered point clouds. Subsequently, the ICP algorithm and the occupation probability algorithm were employed for matching in order to determine the position and orientation of the robot. Finally, a pose optimization method using sparse matrix was utilized to optimize the obtained pose. The critical coefficient was introduced into the TEB algorithm to adjust the distance between the robot and the obstacle, thereby enhancing the safety of the robot’s motion trajectory. The constraints on robot acceleration and terminal velocity improved the smoothness of the robot motion. The experimental results showed that the positioning accuracy of the robot was less than 5 cm, and the angular stability was about 2°. In terms of positioning speed, the improved positioning algorithm was 40% higher than before, effectively reducing the positioning time. Under the guidance of the improved TEB algorithm, the robot is capable of selecting a safer path and moving smoothly. It should be noted that other literature only measures the robot moving a fixed distance and then measuring its error, and ignores the cumulative error in multi-target detection. However, this method cannot guarantee that the robot still has a good positioning accuracy in a complex environment.

The developed inspection robot in this study is capable of intelligently and efficiently performing tasks in complex environments, providing application scenarios for intelligent inspection. However, this research still faces many challenges, such as localization accuracy and speed estimation. Future research will focus on improving the accuracy of robot localization.

## Figures and Tables

**Table 1:** The main parameters of robot motion.

Constraint Parameters	Values
Maximum X linear velocity (m/s)	0.4
Maximum backward linear velocity (m/s)	0.2
Maximum angular velocity (rad/s)	0.4
Maximum X linear acceleration (m/s^2^)	0.3
Maximum angular acceleration (rad/s^2^)	0.3
Obstruction expansion radius (m)	0.5

**Table 2:** Comparison of positioning speed under different maps.

Method	Location Time under 7 m × 3.5 m Map (s)	Location Time under 15 m × 7 m Map (s)
Traditional Method	3.0	4.3
Proposed Method	1.8	2.3

## Data Availability

The data presented in this study are available upon request from the corresponding author. The data are not publicly available due to privacy reasons.
